# Diabetic Ketoacidosis Associated with Sodium-Glucose Cotransporter 2 Inhibitors: Clinical and Biochemical Characteristics of 29 Cases

**DOI:** 10.1155/2023/6615624

**Published:** 2023-07-04

**Authors:** G. A. Stamatiades, P. D'Silva, M. Elahee, G. M. Viana, A. Sideri-Gugger, S. K. Majumdar

**Affiliations:** ^1^Department of Medicine, Yale New Haven Health, Bridgeport Hospital, Bridgeport, CT 06610, USA; ^2^Division of Endocrinology, Diabetes and Hypertension, Brigham and Women's Hospital, Harvard Medical School, Boston, MA 02115, USA; ^3^Division of Rheumatology and Clinical Immunology, University of Pittsburgh, Pittsburgh, PA 15261, USA; ^4^Division of Endocrinology, Columbia University Medical Center, New York, NY 10032, USA

## Abstract

**Objective:**

To describe the clinical and biochemical characteristics of all reported cases of DKA associated with SGLT2 inhibitor use in patients with type 2 diabetes mellitus and to identify potential risk factors.

**Design:**

A retrospective case series was conducted between March 2013 and August 2019 using an electronic medical record search algorithm.

**Results:**

25 patients met the criteria for DKA associated with SGLT2i use (total of 29 cases), 15 were female, average age was 54.24 years, and mean diabetes duration was 8.76 years. The majority of the patients (23 patients) had no history of prior DKA. Average blood glucose concentrations at presentation were 298.9 ± 152.7 mg/dl. Interestingly, nearly half of the episodes (14) met the criteria of euglycemic DKA (glucose <250 mg/dl). Average anion gap values were 26.59 ± 6.15 mg/dl, bicarbonate values were 11.14 ± 5.57 mg/dl, and pH values were 7.16 ± 0.12. All had positive serum and urine ketones. The most common presenting symptoms were nausea, vomiting (18 cases), and abdominal pain (10 cases). Common precipitants were poor oral intake (18 cases) and infection (10 cases). A variety of drugs were prescribed along with an SGLT2i, and 11 of the patients were using insulin. None of the cases were fatal. Comparison between euglycemic DKA and hyperglycemic DKA did not identify any significant difference. A major limitation factor of the study was the lack of control group or comparison to other antiglycemic agents to assess the relative risk.

**Conclusions:**

The majority of SGLT2i-associated DKA cases occurred in patients with T2DM without prior episodes of DKA. The most common presenting symptoms were nausea, vomiting, and abdominal pain, while poor food intake and infection were the main precipitants. Clinicians should consider the possibility of DKA in SGLT2i-treated patients presenting with these symptoms, even in absence of marked hyperglycemia.

## 1. Introduction

Diabetic ketoacidosis (DKA) and glycosuria are defining features of uncontrolled diabetes. Therefore, it is almost paradoxical that agents directed towards the treatment of diabetes would primarily work by provoking glycosuria while raising the risk for DKA. Sodium-glucose cotransporter 2 inhibitors (SGLT2 inhibitors) have shown benefits that extend beyond blood sugar lowering to weight loss, reduced blood pressure, and improved outcomes in heart failure and chronic kidney disease. They were first marketed in 2013 as antihyperglycemic agents for the management of type 2 diabetes mellitus (T2DM), and since then, their use and indications have continued to expand [1]. According to American and European diabetes guidelines, they are now the recommended second-line agents for the management of DM after metformin in patients with chronic kidney disease and heart failure [2].

SGLT2 inhibitors work by blocking glucose reabsorption at SGLT2 channels in the proximal convoluted tubule of the kidney and therefore lower glucose independently of insulin, avoiding hypoglycemia and weight gain, which are associated with insulin or its secretagogues. They are especially appealing given the metabolic benefits of weight loss, improvements in blood pressure, and cardiac and renal benefits independent of diabetes status [3, 4]. However, SGLT2 inhibitors are not necessarily free of harm, and along with their vast benefits, they are also associated with rare but dramatic adverse effects. The most common adverse events identified in clinical trials were genital mycotic and urinary tract infections, but after FDA approval, further adverse events surfaced such as urosepsis, pyelonephritis, Fournier's gangrene, ketoacidosis, and acute kidney injury [5–7]. In 2015, the FDA announced that SGLT2i may lead to ketoacidosis [8]. The FDA Adverse Event Reporting System (FAERS) database from March 2013 to May 2015 identified 73 cases of ketoacidosis in patients with type 1 or type 2 diabetes who had been treated with SGLT2i. In 2016, the American Association of Clinical Endocrinologists and American College of Endocrinology announced a joint position statement on SGLT2i and DKA risk, stating that based on the available data, DKA occurs infrequently, and the benefits of use outweigh the risks [9].

Detecting SGLT2i-associated DKA may be challenging and can be missed or delayed due to an atypical presentation with lower than anticipated glucose levels (<250 mg/dL) termed euglycemic ketoacidosis [9]. Since their use has increased dramatically, identification of patients at greatest risk of this life-threatening complication is essential [10–12]. Therefore, to better understand the circumstances in which SGLT2i-associated DKA occurs, we reviewed all available cases at our hospital over a 5-year period. The primary objective of this study was to describe the clinical and biochemical characteristics of the patients with T2DM who developed DKA and to identify potential risk factors contributing to the incidence of DKA in patients treated with SGLT2i.

## 2. Methods

### 2.1. Study Design

We performed a single-center, retrospective study by review of electronic medical records. The study was approved by our institutional IRB. We identified patients with T2DM using SGLT2i admitted to Yale New Haven Health System's Bridgeport Hospital in Bridgeport, CT, between March 2013 and August 2019 with a diagnosis of DKA using a search algorithm. A comprehensive search was conducted by the investigators using specific drug-related and adverse effect-related combinations of search terms.

### 2.2. Inclusion Criteria

All patients with T2DM using SGLT2i at the time of admission, such as canagliflozin (Invokana), dapagliflozin (Farxiga), empagliflozin (Jardiance), empagliflozin/linagliptin (Glyxambi), empagliflozin/metformin (Synjardy), and dapagliflozin/metformin (Xigduo XR), who developed DKA were included in the study. The DKA diagnosis was made based on the following criteria: pH <7.3, anion gap >12 mmol/L, and presence of ketones in the blood or urine. Patients were further subdivided into euglycemic DKA (euDKA) or hyperglycemic DKA (hDKA) based on the presence of either euglycemia (glucose <250 mg/dl) or hyperglycemia (glucose ≥250 mmg/dl).

### 2.3. Exclusion Criteria

Patients who had DKA with T1DM or had DKA with T2DM but were not treated with SGLT2i were excluded from the study. In addition, patients with anion gap metabolic acidosis who did meet the criteria of DKA were excluded from the study as well.

### 2.4. Data Extraction

From the identified cases, both subject characteristics (e.g., age, sex, average BMI, and type of DM) and characteristics related to the DKA episode (e.g., laboratory values, symptoms, and precipitating factors) were collected.

### 2.5. Statistical Analyses

Descriptive and summary statistics were used to describe the study cohort's clinical and biochemical characteristics. Continuous variables were presented as means (±SD), while categorical variables were presented as absolute values and percentages. Pairwise comparisons between continuous and categorical variables were carried out with *t*-test and chi-square tests, respectively.

## 3. Results

**Figure 1:**
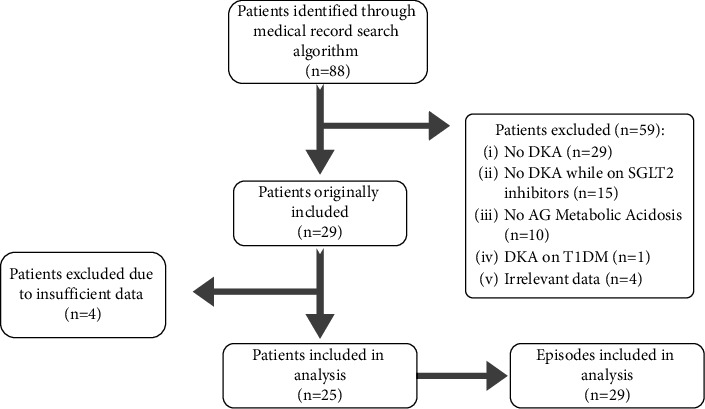
Search term results.

Twenty-five patients were included, representing 29 episodes of DKA. The initial search identified 88 patients, but only 29 patients were initially included in the study, and the rest of the 59 patients were excluded as they did not meet the criteria. Twenty-nine patients had no DKA, 15 patients had DKA but were not on an SGLT2i, 10 had non-anion gap metabolic acidosis, 1 patient had DKA on SGLT2i but had T1DM, and 4 were associated with irrelevant data. Of the 29 patients identified that met inclusion criteria, 4 were excluded due to insufficient data (Figure 1).

Of the 29 episodes of DKA included in the study, 12 involved empagliflozin, 10 involved canagliflozin, and 7 involved dapagliflozin. Twenty-three patients had no history of DKA prior to using an SGLT2i. Fifteen patients were female (60%) and 10 (40%) were male. The average age at presentation was 54.24 years, average BMI was 30.64 kg/m^2^, and mean diabetes duration was 8.7 years. Other demographic characteristics were collected and are summarized in Table 1. A variety of drugs were prescribed along with an SGLT2i for patients with T2DM, and eleven patients (37.9%) were using insulin.  Table 2 summarizes the medications used along with SGLT2i.

The majority of the patients presented with nausea and vomiting (18 patients, 62.07%) and abdominal pain (10 patients, 34%). Other presenting features included shortness of breath (6 patients, 20.69%), altered mental status (4 patients, 13.79%), fatigue (4 patients, 13.79%), and fever and chills (3 patients, 10%) (Table 3). The average blood glucose concentration at presentation was 298.9 ± 152.7 mg/dl. Interestingly, fourteen of the episodes (48.2%) were euglycemic DKA (euDKA) with blood glucose <250 mg/dl. Average anion gap values were 26.59 ± 6.15 mg/dl, bicarbonate values were 11.14 ± 5.57 mg/dl, and pH values were 7.16 ± 0.12. All had positive urine ketones, and average beta-hydroxybutyrate levels were 6.71 ± 3.94 mmol/l. Average hemoglobin A1c was 9.71 ± 3% (Table 4).

Potential precipitating factors were poor oral intake (18 patients, 62%) and infection (10 patients, 34.48%). Other noted precipitants were acute kidney injury, myocardial infarction, pancreatitis, and surgery (Table 5). In our study, no significant difference was identified in the precipitating factors or clinical presentation of patients presenting with euDKA or hDKA. All the patients were hospitalized for treatment of the DKA episode, and 25 cases required treatment with intravenous insulin. The rest of the cases were treated with subcutaneous insulin. None of the cases were fatal. SGLT2 inhibitors were discontinued after hospital discharge, and they were included in the allergy list.

## 4. Discussion

The American Diabetes Association characterizes diabetic ketoacidosis by a triad of uncontrolled hyperglycemia, metabolic acidosis, and increased total body ketones [13]. Despite the advances in the understanding of pathophysiology and therapy, this complication tends to be a source of significant morbidity and mortality in patients with diabetes. According to the CDC, hospitalizations for DKA declined slightly from 2000 to 2009 and then again began to rise by >6% annually from 2009 to 2014 [14]. The aggregate national cost of treating DKA in 2003 was 2.2 billion which increased to 5.1 billion in 2014 [15].

SGLT2 inhibitors can favor the development of DKA in several ways. They are known to increase renal absorption of ketone bodies causing ketonemia with normal plasma glucose levels [16, 17]. When SGLT2 inhibition produces glycosuria, the resulting reductions in blood glucose levels are associated with decreased insulin and increased glucagon secretion promoting a shift towards lipid oxidation and ketogenesis. SGLT2 inhibitors also appear to increase renal *β*‐oxidation resulting in increased ATP synthesis which in turn decreases renal *β*-hydroxybutyrate oxidation, a source of bicarbonate. Thus, in the presence of a developing acidosis, the reduced production of bicarbonate limits normal buffering capacity and the defense against systemic acidosis. Taken together, in the setting of increased ketone production, serum ketone levels rise contributing to acidosis, while decreased renal ketone oxidation and losses in the urine result in decreased buffering with the net effect being an increased susceptibility for ketoacidosis [16–18].

In a systematic review and meta-analysis of RCTs conducted by Liu et al., the risk of DKA was higher in patients with diabetes on SGLT2i than controls. Thirty-nine RCTs were included, involving 60,580 patients and 85 episodes of DKA. DKA events were approximately 2-fold greater in SGLT2 treated vs. comparator patients, 0.18% vs. 0.09%, respectively. Larger relative effects were seen in patients over 60 years of age and people on SGLT2i for >52 weeks [19]. Additionally, Fralick et al. showed that the risk of developing DKA within 180 days of initiation of an SGLT2i was twice that of DPP-4 inhibitors [20]. A review conducted by Menghoum and Hermans who investigated clinical and biochemical characteristics of euglycemic DKA in patients with T2DM while on SGLT2i found that the average age at presentation was 57.6 ± 14.9 years, 48.6% were female, 52.8% were male, duration of DM was roughly 12.2 ± 9.9 years, average BMI was 27.6 ± 6.2 kg/m^2^, and euglycemic DKA occurred at different times of usage of SGLT2 inhibitors [21]. In our study, 60% were female and 40% were male, average age was 54.24 years, average BMI was 30.64 kg/m^2^, and mean diabetes duration was 8.7 years. The mean time that DKA occurred after SGLT2i initiation was 337 days (range <7 days to > 3 years).

Despite numerous reports stating that the vast majority of DKA cases occur in patients with type 1 DM, nearly all of the cases (except one) initially identified consisted of patients with a diagnosis of type 2 DM. However, a large portion of cases of SGLT2 inhibitor-associated DKA have occurred in individuals with diabetes who are insulin deficient (11 patients, 37.9% of the cases). Some patients in our study may have had ketosis-prone diabetes, undiagnosed T1DM, or latent autoimmune diabetes in adults (LADA) and may have been misclassified as T2DM. In a population-based cohort study conducted by Douros et al. which included data from 7 Canadian provinces and the United Kingdom, it was seen that compared to DPP-4 inhibitors, SGLT2 inhibitors were associated with an increased risk of DKA (HR: 2.85) (95% CI: 1.99–4.08); however, interestingly, the compound-specific hazard ratios were 1.86 for dapagliflozin, 2.52 for empagliflozin, and 3.58 for canagliflozin [22]. In our study, 10 of the 30 episodes of DKA involved patients on canagliflozin and only 7 involved dapagliflozin.

DKA may be difficult to recognize when glucose levels are not elevated to the extent where DKA is normally defined (>250 mg/dL) [23]. When Blau et al. reviewed the data reported by the FDA Adverse Event Reporting System, it was noted that most of the cases (71%) of DKA reported with SGLT2 inhibitors were euglycemic DKA [24]. In another multicohort study, 58% of the cases met the criteria of euglycemic DKA [25]. In our study, almost half of the episodes were euDKA. Our DKA cases are all of them in an association with the common them of medication and lack of prior DKA history (this was the first episode of DKA in 92% of the patients) and patients who are typically not at risk for DKA. Our study provides data on a spectrum of glucose values associated with these medicines and DKA. Perhaps by limiting glucose to 250 mg/dL as a threshold, previous report may have missed the extent of DKA cases associated with these drugs. There is no clear reason why all patients would need to have glucose values less than 250 mg/dL. In the setting of hypovolemia, and decreased urine output, the rates of glucose increase might exceed the ability of SGLT2i to lower glucose enough, yet the effect on ketone production would still be present and contribute to acidosis. Some patients might have greater liver glycogen stores at baseline than others, and this too could possibly explain why one might have higher vs. lower glucose values in the setting of SGLT2i-associated DKA.

Our study identified several factors that may help physicians detect DKA early on even in the absence of profound hyperglycemia. Sixty-two percent of the patients in our study presented with complaints of nausea and vomiting, and 34% of them had abdominal pain. Other clinical features seen included shortness of breath, fatigue, and altered mental status. In a study conducted by Seth et al. in India examining the clinical profile of patients with DKA, 63.3% of their patients had nausea and vomiting, abdominal pain was present in 43.3% of the patients, and altered mental status was present in 30% of the cases [26]. It was also noted that 33% of the patients were dehydrated [26]. Similarly, in another study by Munro et al., which evaluated the clinical characteristics of patients with euDKA, patients mainly complained of nausea and vomiting (86%), abdominal pain (27%), and polyuria/polydipsia (24%) [27]. The clinical features in both studies are similar to those seen in our study, and while generally nonspecific, when nausea, vomiting, and abdominal pain are present in combination with the use of an SGLT2i and evidence for acidosis, a diagnosis of DKA should be considered despite glucose levels less than 250 mg/dL.

The risk for SGLT2i-associated DKA appears to be increased under certain circumstances. A systematic review found that the most common triggers for euDKA were prolonged fasting (24.4%), acute surgical intervention (20.8%), acute infection (16.7%), relative insulinopenia or a recent decrease/cessation of insulin therapy (14.2%), and dehydration (9.2%) [21]. Another systematic review conducted by Burke et al. found increased risk among patients diagnosed with T2DM who were eventually found to have LADA, those recently undergoing major surgery, and patients who had recently decreased or stopped insulin [7]. In our study, major precipitating factors were poor oral intake (62%) and reduction in insulin dose (54.5% of those using insulin). Other precipitants were severe infections, surgery, myocardial infarction, and acute kidney injury. Almost all cases are associated with metabolically stressful events, indicating that when the delicate balance between insulin and glucagon is lost, ketoacidosis occurs.

Data from this study and previous studies should be helpful for recognizing those at risk for SGLT2i-associated DKA and what features should prompt its consideration. The risk for DKA is increased by circumstances of stress, such as infection, acute illness (e.g., MI), and surgery, often combined with fasting or poor nutritional intake. Under these conditions, ketones naturally rise and glycosuria can lead to lower glucose levels. Nausea, vomiting, and abdominal pain are common manifestations of DKA, and their presence in someone on an SGLT2i should prompt its consideration. However, our study did not notice any significant differences in the precipitating or clinical features that would help differentiate DKA from those presenting with euDKA. Though we are unable to differentiate based on symptomatology, the overall aim is to reduce the risk of DKA irrespective of the presenting glucose levels. Having accurate diagnostic information on patients with diabetes prior to prescribing SGLT2i could be helpful. For example, screening select patients for prior episodes of DKA, and checking antibodies and c-peptide levels, might uncover diagnoses consistent with ketosis-prone diabetes, LADA, or T1DM. The assessment of *β*-cell functionality in patients who may have progressed to relative insulin deficiency such as those with longstanding T2DM would be another susceptible group to evaluate. Assessing baseline fasting ketone levels prior to the initiation of SGLT2i therapy may have a role in identifying those more prone to ketoacidosis, but further studies are warranted. While the presence of diagnoses such as LADA or longstanding T2DM where insulin therapy is needed does not necessarily preclude patients from using SGLT2i, they should at least prompt judicious counseling and monitoring.

Our study has several limitations. Although a comprehensive search was conducted, not all cases may have been identified. The collected data are from a small sample size which makes it difficult to generalize conclusions to the entire population of diabetic patients treated with SGLT2i. In addition, four cases were excluded due to insufficient data limiting biochemical data. We did not investigate the risk for DKA with other oral agents to compare the relative risk. In addition, though our cases were seen in those classified as T2DM, insulin antibodies were checked only in 8 patients (one of whom was GAD65 positive) and C-peptide was checked in only 7 patients and during the acute phase of DKA where we would expect to see a low C-peptide. Thus, there is a possibility that repeating c-peptide levels and insulin antibodies would help uncover patients with T1DM or LADA or ketosis-prone diabetes that were misclassified as Type II. In the end, while DKA can occur in T2DM under severe stress, these cases highlight its unexpected development in patients who had no prior history of DKA and would typically not be thought of as high risk for DKA under their presenting circumstances. Therefore, an association still exists between SGLT2i use and all of these cases, but, similar to other case series, it is not possible to prove causation without a randomized study that reproduces the circumstances in which DKA developed. Instead, this case series helps to broaden the glycemic context in which DKA is associated with SGLT2i use beyond that of an arbitrary glucose threshold of 250 mg/dL. The reported clinical and biochemical characteristics are consistent with the previously published systematic reviews. These findings may help clinicians identify DKA events promptly and prevent progression to more severe DKA, which can ultimately improve patient care and reduce healthcare costs.

## 5. Conclusion

Our study found that the majority of SGLT2i-associated DKA cases occurred in patients with T2DM without prior episodes of DKA. The most common presenting symptoms were nausea, vomiting, and abdominal pain, while poor food intake and infection were the main precipitants. Clinicians should consider the possibility of ketoacidosis in SGLT2i-treated patients presenting with these symptoms, even in absence of marked hyperglycemia. If DKA is diagnosed, the SGLT2i should be discontinued and insulin therapy for DKA should be initiated. Furthermore, establishing appropriate guidance around the use of SGLT2i's during the aforementioned high-risk situations would be an important step in the prevention of SGLT2i-associated DKA.

## Figures and Tables

**Table 1:** Baseline characteristics.

Baseline characteristics	Total DKA	EuDKA	hDKA
Number of cases, *N* (%)	29 (100%)	14 (48.2%)	15 (51.8%)
Gender, *N* (%)			
Male	10 (40%)	7 (50%)	4 (30.8%)
Female	15 (60%)	7 (50%)	9 (69.2%)
SGLT2i agent, *N* (%)			
Canagliflozin	10 (34.4%)	3 (21.4%)	7 (46.6%)
Empagliflozin	12 (41.3%)	7 (50%)	5 (33.3%)
Dapagliflozin	7 (24.1%)	4 (28.6%)	3 (20.1%)
Duration of diabetes, mean + SD, years	8.76 ± 5.73	9.08 ± 6.4	8.46 ± 5.28
Prior DKA, *N* (%)			
Yes	2 (8%)	2 (14.2%)	2 (15.4%)
No	23 (92%)	12 (85.8%)	11 (84.6%)
Age, mean ± SD, years	54.24 ± 12.41	55.64 ± 11.65	52.93 ± 13.3
Average BMI, mean ± SD, kg/m^2^	30.64 ± 6.29	29.86 ± 5.46	31.37 ± 7.09
A1c, mean ± SD, %	9.71 ± 3.05	9.16 ± 3.1	10.19 ± 3.03

One male patient had one episode of euDKA and one episode of hDKA, and one female patient had one episode of euDKA and three episodes of hDKA.

**Table 2:** Medication regimens.

Medication regimen, *N* (%)	Total DKA	EuDKA	hDKA
SGLT2i only	0 (0%)	0 (0%)	0 (0%)
SGLT2i + metformin	1 (3.45%)	0 (0%)	1 (6.7%)
SGLT2i + thiazolidinedione	1 (3.45%)	1 (7.1%)	0 (0%)
SGLT2i + GLP1 agonist	1 (3.45%)	1 (7.1%)	0 (0%)
SGLT2i + sulfonylurea	1 (3.45%)	0 (0%)	1 (6.7%)
SGLT2i + 2 agents	10 (34.5%)	5 (35.7%)	5 (33.3%)
SGLT2i + ≥3 agents	4 (13.8%)	3 (21.5%)	1 (6.7%)
SGLT2i + insulin ± other agents	11 (37.9%)	4 (28.6%)	7 (46.6%)

**Table 3:** Symptoms on presentation.

Symptoms, *N* (%)	Total DKA	EuDKA	hDKA
Nausea, vomiting	18 (62.07%)	10 (71.42%)	8 (53.33%)
Abdominal pain	10 (34%)	5 (35.71%)	5 (33.33%)
Shortness of breath	6 (20.69%)	0 (0%)	6 (40%)
Altered mental status	4 (13.79%)	1 (7.14%)	3 (20%)
Fatigue	4 (13.79%)	3 (21.42%)	1 (6.66%)
Fever, chills	3 (10%)	3 (21.42%)	0 (0%)

**Table 4:** Biochemical parameters on presentation.

Laboratory value, mean ± SD	Total DKA	EuDKA	hDKA
Blood glucose (mg/dl)	298.9 ± 152.7	187.92 ± 33.93	402.53 ± 147.43
Carbon dioxide (mEq/l)	11.14 ± 5.57	12.43 ± 4.4	9.93 ± 6.4
Anion gap (mEq/l)	26.59 ± 6.15	25.57 ± 4.12	27.53 ± 7.61
pH	7.16 ± 0.13	7.22 ± 0.1	7.11 ± 0.13
Beta-hydroxybutyrate (mmol/l)	6.71 ± 3.94	5.59 ± 2.41	7.57 ± 4.75
Lactate (mmol/l)	1.73 ± 0.75	2.55 ± 0.56	2.04 ± 0.85
Creatinine (mg/dl)	1.1 ± 0.94	0.91 ± 0.29	1.27 ± 1.28
BUN (mg/dl)	20.38 ± 14.28	20.35 ± 7.75	20.4 ± 18.76

**Table 5:** Precipitating factors.

Precipitating factor, *N* (%)	Total DKA	EuDKA	hDKA
Poor oral intake	18 (62.07%)	9 (64.28%)	9 (60%)
Infection	10 (34.48%)	6 (42.85%)	4 (26.66%)
Acute kidney injury	2 (6.9%)	0 (0%)	2 (13.33%)
Myocardial infarction	1 (3.45%)	1 (7.14%)	0 (0%)
Pancreatitis	1 (3.45%)	0 (0%)	1 (6.66%)
Surgery	1 (3.45%)	1 (7.14%)	0 (0%)
Reduction of insulin	6/11 (54.55%)	3/4 (75%)	3/7 (42.85%)

## Data Availability

The patients' clinical and biochemical data used to support the findings of this study are available from the corresponding author upon request.
